# Complete genome sequence of *Paenibacillus* sp. VCA1 isolated from crater lake of the El Chichón Volcano

**DOI:** 10.1128/MRA.00583-23

**Published:** 2023-10-26

**Authors:** Nancy Abril Martínez-López, Betsy Anaid Peña-Ocaña, Rodolfo García-Contreras, Toshinari Maeda, Reiner Rincón-Rosales, Adrián Cazares, Yuki Hoshiko, Víctor Manuel Ruíz-Valdiviezo

**Affiliations:** 1 Laboratorio de Biología Molecular, Tecnológico Nacional de México/Instituto Tecnológico de Tuxtla Gutiérrez, Carretera Panamericana ,Tuxtla Gutiérrez, Chiapas, México; 2 Departamento de Bioquímica, Instituto Nacional de Cardiología, Juan Badiano, Colonia Sección XVI, Tlalpan, Mexico City, Mexico; 3 Departamento de Microbiología y Parasitología, Facultad de Medicina, Universidad Nacional Autónoma de México, Mexico City, México; 4 Department of Biological Functions Engineering, Kyushu Institute of Technology, Kitakyushu, Fukuoka, Japan; 5 EMBL's European Bioinformatics Institute (EMBL-EBI), Wellcome Genome Campus, Cambridge, Massachusetts, United Kingdom; 6 Wellcome Sanger Institute, Wellcome Genome Campus, Cambridge, Massachusetts, United Kingdom; 7 Division of Microbiology, Department of Infectious Medicine, Kurume University School of Medicine, Asahi-Machi, Kurume-City, Fukuoka, Japan; Rochester Institute of Technology, Rochester, New York, USA

**Keywords:** biocontrol, extremophile bacteria, extreme environments

## Abstract

We report the complete genome of *Paenibacillus* sp. strain VCA1, which was isolated from sediment from El Chichón Volcano. This genome consists of 6,690,819 bp and 6,312 coding sequences, with 51.8% G+C content. Whole-genome sequencing was performed to explore the strain’s biocontrol and plant growth promotion properties.

## ANNOUNCEMENT

In recent years, microorganisms from diverse extreme environments have been studied, and several genera of bacteria (1) capable of adapting and living in hostile conditions have been identified. One the predominant genera in this category is the genus *Paenibacillus* (2), which is a bacterium capable of producing metabolites, and developing mechanisms are involved in the promotion of plant growth such as the production of phytohormones, phosphate solubilization, and nitrogen fixation; the biocontrol of phytopathogens by producing lytic enzymes; and the biosynthesis of antimicrobials (3 – 7).

*Paenibacillus* sp. VCA1 strain was isolated from the volcanic sediment. The sediment was collected from the shore of the lake (17°21′40″N, 93°13′40″W). A sample was taken in triplicate from the surface layer of the sediment (15 cm depth) at a temperature of 81°C. For bacterial cultures, 1 g of sediment was suspended in 10 mL of volcanic water (collected from the same sampling point) and left in agitation at 120 rpm and at 50°C for 12 h. Subsequently, 1 mL of the suspension was taken and inoculated in 10 mL of Luria Bertani (LB) liquid culture medium and then incubated at 50°C for 72 h. The selection of isolates obtained from the liquid cultures was based on the morphological diversity of their colonies (8). Isolation of strain VCA1 was performed on LB agar by using the cross-streak plate technique until the pure culture was obtained and incubated at 50°C for 72 h. This bacterium was tested positive for biocontrol against the phytopathogen *Fusarium oxysporum* 45ta by use of the dual confrontation technique, where by potato dextrose agar (PDA ) medium was used and incubations occurred at 28°C for 7 days (9, 10)

**Fig 1:**
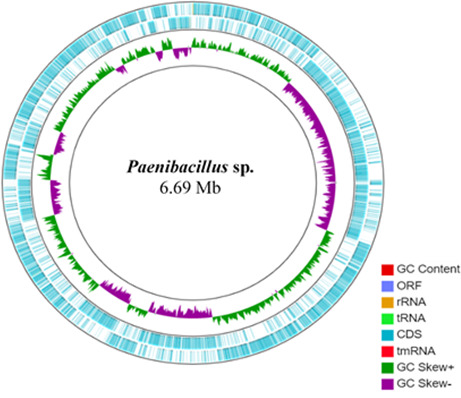
Circular map of the complete genome of *Paenibacillus* sp. The map was made with GC view server.

Total genomic DNA was extracted from the overnight culture by using the DNeasy UltraClean Microbial Kit (Qiagen Inc., Valencia, CA, USA). The concentration of the extracted DNA was determined by using a NanoDrop spectrophotometer and a Qubit 2.0 Fluorometer (Invitrogen, Qubit-ITTM dsDNA HS Assay Kit). Subsequently, genomic DNA libraries were created with the Nextera XT DNA Library Preparation Kit (Illumina, San Diego, CA, USA). These libraries were then subjected to sequencing on an Illumina MiSeq system, thus generating 301-bp paired-end reads. Quality of readings was assessed with FastQC v.1.0.1, whereas low-quality readings and sequence adapters were removed with Trimmomatic v0.36. Genome assembly was performed with SPAdes from the KBase Predictive Biology platform v3.15.3 (11, 12). Genome assembly yielded a genome sequence of 6,690,819 bp, with a G + C content of 51.8%. A total of 88 contigs were obtained that had N_50_ value of 483,213. Genomic map construction was done with the GCview program in Java software v.11 (Fig. 1). The contigs were annotated using the Rapid Annotations Subsystems Technology (RAST) v2.0 Toolkit. A total of 6,312 coding genes, 324 subsystems, and 99 RNAs genes were identified. Genome annotation was performed with RAST-The Seed Server in conjunction with PROKKA v1.15.5 (12). We found 3, 29, and 5 genes involved in isoprenoid, N-acetylglucosamine, and siderophore production, respectively. These secondary metabolites are involved in plant growth promotion and phytopathogens biocontrol (13 – 15). Taxonomic assignment was carried out via tetranucleotide similarity (TETRA) and average nucleotide identity (ANIb and ANIm) by using the Jspecies tool and DNA-DNA hybridization with the genome-genome distance calculator (GGDC) method on the Deutsche Sammlung von Mikroorganismen und Zellkulturen(DSMZ) server (16), which showed similarity to *Paenibacillus* sp. strain P1XP2 (GenBank accession number txid1472719).

## Data Availability

This whole genome shotgun project has been deposited at GenBank under the accession number JAVLSX000000000.1. The sequenced genome is publicly available under SRA, BioProject, and BioSample numbers, which are SRR25304438, PRJNA993667, and SAMN33190539, respectively. This genome is publicly available. The version described in this paper is the first version.
